# BRAF V600E Status and Stimulated Thyroglobulin at Ablation Time Increase Prognostic Value of American Thyroid Association Classification Systems for Persistent Disease in Differentiated Thyroid Carcinoma

**DOI:** 10.1155/2019/3081497

**Published:** 2019-04-07

**Authors:** Andrea Repaci, Valentina Vicennati, Alexandro Paccapelo, Ottavio Cavicchi, Nicola Salituro, Fabio Monari, Dario de Biase, Giovanni Tallini, Annalisa Altimari, Elisa Gruppioni, Michelangelo Fiorentino, Uberto Pagotto

**Affiliations:** ^1^Endocrinology Unit, Sant'Orsola-Malpighi Hospital, University of Bologna, Bologna, Italy; ^2^Department of Otolaryngology, Sant'Orsola-Malpighi Hospital, University of Bologna, Bologna, Italy; ^3^Radiotherapy Unit, Sant'Orsola-Malpighi Hospital, University of Bologna, Bologna, Italy; ^4^Department of Pharmacy & Biotechnology (Dipartimento di Farmacia e Biotecnologie, FaBit) - Molecular Diagnostic Unit, Azienda USL di Bologna, University of Bologna, Bologna, Italy; ^5^Department of Medicine (Dipartimento di Medicina Specialistica, Diagnostica e Sperimentale) - Molecular Diagnostic Unit, Azienda USL di Bologna, University of Bologna School of Medicine, Bologna, Italy; ^6^Pathology Unit, Department of Experimental, Diagnostic and Specialty Medicine (DIMES), S.Orsola-Malpighi Hospital, University of Bologna, Bologna 40138, Italy

## Abstract

**Background:**

Stimulated thyroglobulin levels measured at the time of remnant ablation (A-hTg) and BRAF^V600E^ mutation had shown prognostic value in predicting persistent disease in differentiated thyroid cancer (DTC). The aim of this study was to evaluate the prognostic role of A-hTg combined with the BRAF^V600E^ status in association with the revised American Thyroid Association (ATA) risk stratification.

**Material and Methods:**

620 patients treated for a DTC were included in this study with a median follow-up duration of 6.1 years. All patients underwent total thyroidectomy followed by radioiodine ablation. Patients with positive anti-thyroglobulin antibodies were excluded. The predictive value of A-hTg was calculated by receiver operating characteristic curve (ROC curve) analysis. The Cox proportional hazard regression model, including the *BRAF* status, A-hTg, and ATA classification system, was assessed to evaluate the existing persistent disease risk.

**Results:**

Taken together, the *BRAF* status and A-hTg levels improve the ATA risk classification in all categories. In particular, in the low-risk ATA classification, only the combination of BRAF^V600E^+A-hTg > 8.9ng/ml was associated with persistent disease (*P* = 0.001, HR 60.2, CI 95% 5.28-687). In the intermediate-risk ATA classification, BRAF^WT^+A-hTg > 8.9ng/ml was associated with persistent disease (*P* = 0.029, HR 2.71, CI 95% 1.106-6.670) and BRAF^V600E^+A-hTg > 8.9ng/ml was also associated with persistent disease (*P* < 0.001, HR 5.001, CI 95% 2.318-10.790). In the high-risk ATA classification, both BRAF^V600E^+A-hTg < 8.9ng/ml and BRAF^V600E^+A-hTg > 8.9 ng/ml were associated with persistent disease (*P* = 0.042, HR 5.963, CI 95% 1.069-33.255 and *P* = 0.002, HR 11.564, CI 95% 2.543-52.576, respectively).

**Conclusions:**

The *BRAF* status and stimulated thyroglobulin levels at ablation time improve the ATA risk stratification of differentiated thyroid cancer; therefore, even A-hTg could be included in risk classification factors.

## 1. Introduction

The papillary thyroid carcinoma (PTC) is the most common epithelial thyroid tumour, representing approximately 80% of all thyroid cancers [1]. Although the incidence of thyroid tumours has significantly increased over the past three decades [2], the prognosis remains unchanged over time, with overall 10 yr survival rates higher than 90-95% [3]. About 5-10% of PTC patients develop regional or local recurrences, 10-15% have a persistent disease (PD), and 10-15% develop distant metastases with an overall 10 yr survival rate of 40% [4]. Among oncogene alterations involved in the pathogenesis of PTC, v-raf murine sarcoma viral oncogene homolog BRAF^V600E^ is the most frequent mutation [5]. In the literature, there are conflicting data about the impact of BRAF^V600E^ on prognosis and mortality [6–13]. Some authors proposed the inclusion of BRAF mutation in the ATA risk classification in order to better stratify the recurrence risk [14, 15].

Another well-known factor involved in the prediction of persistent disease (PD) is the stimulated serum thyroglobulin (hTg) level measured at radioiodine ablation. Recently, some authors have proposed a value of ablation hTg (A-hTg) lower than 10 ng/ml as a favourable factor on the subsequent disease-free status [16]. Additionally, different retrospective studies have confirmed this predictive prognostic value [17–19].

To date, only Kim et al. have demonstrated that the combination of stimulated A-hTg and the ATA staging system could discriminate the prognosis of patients with DTC after radioiodine ablation [20]. To the best of our knowledge, no studies have evaluated the role of *BRAF* mutations together with stimulated A-hTg with respect to the ATA staging system in order to predict PD. The aim of our study was to retrospectively analyse the impact of *BRAF* mutations and stimulated A-hTg levels at ablation time on the clinical outcome in a monocentric cohort of 620 patient [20].

## 2. Material and Methods

### 2.1. Case Selection

This is a retrospective study involving 620 patients with PTC, followed at the Endocrinology Unit of S.Orsola-Malpighi Hospital from 2000 to 2016. All patients had positive cytology for suspected PTC and underwent total thyroidectomy with lateral neck and central compartment dissection in the case of presurgical evidence of lymph nodes metastases (LNM). The AJCC 7th edition was used to define anatomopathological staging. After surgery, 555 patients underwent radioiodine ablation with doses ranging from 30 to 100 mCi: 316 after stimulation with endogenous TSH (suspension of LT4 for at least 3-4 weeks, thyroid hormone withdrawal (THW)) and 235 after rhTSH administration (Thyrogen®). In patients who suspended LT4, thyroglobulin was assessed on the same day as radioiodine administration (Ablation Thyroglobulin (A-hTg)); in patients treated after rhTSH stimulation, thyroglobulin was assessed in basal conditions and five days after the first Thyrogen injection (A-hTg). A whole-body scan (WBS) was performed 5-7 days after the radioiodine administration. Clinical and anatomopathological data were collected in an electronic database. Patients were classified into two groups according to the *BRAF* molecular status: “group 1” consisted of patients without *BRAF* mutation (BRAF^WT^), while “group 2” consisted of those with *BRAF* p.V600E (c.1799T>A) mutation (BRAF^V600E^).

Follow-up was scheduled every 6 to 12 months, by dosing thyroglobulin (hTg) on LT4-suppressive therapy (TSH < 0.01 mcUI/ml) or after endogenous stimulation (off LT4 for 3-4 weeks since 2003), or after stimulation by recombinant human TSH (rhTSH) administration, plus neck ultrasound or diagnostic whole-body scan (WBS) and/or other imaging procedures such as CT and/or FDG-PET, when necessary. The study has been approved by the local ethics committee.

### 2.2. Definition of Remission, Persistence, and Recurrence of Disease and Delayed Risk Stratification

Complete remission was defined by the combination of (i) undetectable hTg levels during suppressive therapy or <1 ng/ml after THW or with rhTSH (Thyrogen®) in the absence of anti-Tg antibodies (Ab antiTg) and (II) absence of metastases at neck ultrasound and/or diagnostic WBS or other imaging techniques (CT scan and PET-FDG).

Persistent disease (PD) was defined by (i) stimulated hTg > 1 ng/ml and (ii) positive neck ultrasound and/or diagnostic WBS or other imaging techniques (CT scan and PET-FDG). Recurrence was defined, according to the National Cancer Institute (https://www.cancer.gov/dictionary), as cancers that occur either in the same location as the primary tumour or locally or remotely after a period of observation in which the tumour was no longer identifiable.

Delayed risk stratification (DRS) was defined 8-12 months after ablation as (i) negative, when unstimulated hTg levels were undetectable, or when stimulated hTg was <1 ng/ml with negative Ab antiTg, and negative neck ultrasound and/or diagnostic WBS or other imaging techniques (CT scan and PET-FDG) (negative DRS group), and (b) positive, when stimulated hTg was ≥1 ng/ml, or neck ultrasound and/or diagnostic WBS or other imaging techniques (CT scan, PET-FDG) were positive for disease recurrence.

### 2.3. DNA Extraction and *BRAF* Analysis

Ten *μ*m thick sections from representative Formalin-Fixed, Paraffin-Embedded (FFPE) blocks of papillary thyroid carcinoma were used. Blocks with the highest enrichment of tumour cells over stroma, inflammation, or normal thyroid tissue were selected by a pathologist (M.F.) on macroscopically circled and scalpel-dissected haematoxylin and eosin slides. Tumour-cell enrichment was expressed as the percentage of neoplastic over the total number of nuclei in the selected area.

Genomic DNA was extracted using the QIAamp DNA Micro Kit (Qiagen, Hilden, Germany), according to manufacturer instructions with overnight proteinase K digestion and eluted in a 50 *μ*l volume in water. The concentration of the extracted DNA was assessed by real-time PCR using the Quantifiler Kit (Life Technologies, Foster City, CA). All genomic DNA was stored at -20C° until used.

### 2.4. *BRAF* Mutation Analysis

Mutation analysis in the oncogene *BRAF* (exon 15) was performed using the direct Sanger sequencing method [21]. After PCR reaction, amplified DNA was purified using the MinElute PCR purification kit (Qiagen, Hilden, Germany), then visualized and quantified after electrophoresis on 2% agarose gel. Sequencing was carried out using the BigDye Terminator sequencing kit v.3.0 (Life Technologies, Foster City, CA). Sequencing analysis was performed using an automated sequencer (3730xl DNA Analyzer, Life Technologies), and the results were interpreted with the Chromas Software version 1.45 (Technelysium, Australia).

### 2.5. Chemical Assays

The hTg levels were assessed using a solid-phase immunochemiluminometric assay (ICMA) with a functional sensitivity of 0.9 ng/ml (IMMULITE 2000; Diagnostic Products Corp., Los Angeles, CA). To avoid misinterpretation of the Htg measurements, patients were routinely screened for Ab antiTg by electrochemiluminescence immunoassay (ECLIA) with imprecision of <8% for AbTg and <10% for anti-thyroperoxidase antibodies (IMMULITE 2000). The TSH measurement was performed using the IMMULITE 2000 ECLIA (imprecision < 5%).

### 2.6. Statistical Analysis

Data are reported as means ± standard deviations (SD), medians, ranges, and frequencies. The hazard ratios (HRs) were computed together with their 95% confidence intervals (95% CIs). Fisher's exact test, chi-square test, and linear-by-linear association were used to evaluate the associations and differences in the clinical and pathological settings between the two BRAF groups. A ROC (receiver operating characteristic) curve was used to calculate the cut-off value of A-hTg in all patients and in patients prepared by THW or Thyrogen matching with persistent disease. We used the asymptotic *Z*-test to compare the area under the ROC curve (ROC-AUC) of A-hTg in all patients and in patients prepared by THW or Thyrogen.

Cox regression was used as a multivariate analysis to identify the risk factors associated with PD based on the last follow-up date. Kaplan-Meier curves were used to plot the PD with regard to the *BRAF* status in combination with A-hTg in all patients and according to the ATA risk classification. Statistical analyses were performed with SPSS version 15. A two-tailed *P* value less than 0.05 was considered statistically significant.

## 3. Results

### 3.1. Clinical Pathological Features and Main Outcomes according to BRAF Status

BRAF^V600E^ mutation was found in 57.6% of the patients. As reported in Table 1, the BRAF^V600E^ group included more aggressive PTCs: indeed, they more frequently underwent radioiodine treatment compared to the BRAF^WT^ group; moreover, PTC BRAF^V600E^ received higher therapeutic radioiodine doses than the BRAF^WT^ cohort. Recurrence, as well as DRS and PD, was significantly more frequent in the BRAF^V600E^ group. In addition, we observed a strong association between the DRS status and PD: the DRS status, defined 12 months after radioiodine treatment, showed a good sensitivity of 86.1% and specificity of 94.3% in predicting PD at the last follow-up (*P* = 0.001).

We also found a negative association between distant metastases and mutated *BRAF* tumours. Patients with distant metastases are often BRAF^WT^. These data agreed with other studies showing that the *BRAF* mutation is not enough for the development of distant metastases.

### 3.2. Clinical Predictors of PD in PTC Patients

As reported in Table 2, Cox regression analysis for PD found that A-hTg > 8.9 ng/ml, BRAF^V600E^ mutation, tumour dimension (T), lymph node involvement (N), and microscopic surgical margins (R) were significantly associated with PD.

### 3.3. A-hTg Cut-Off

**Figure 1:**
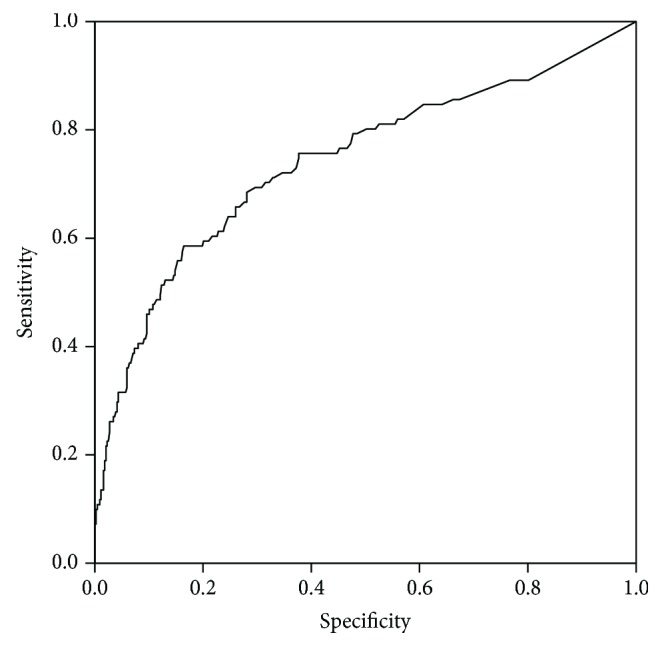
ROC curve of A-hTG in PTC patients.

A ROC curve (crossing A-hTg with PD) demonstrated that stimulated A-hTg, after either rhTSH or THW, was predictive of PD at the last follow-up. The global cut-off value of the A-hTg level was established at 8.9 ng/ml, with a sensitivity of 83.5%, specificity of 58.6%, and AUC of 0.677 (Figure 1). No significant difference was observed regarding stimulated A-hTg prepared by THW (32.5 ± 223) or Thyrogen (44.5 ± 307) (*P* = 0.600). There were no significant differences between ROC-AUC of stimulated A-hTg prepared by THW and ROC-AUC of stimulated A-hTg after Thyrogen (data not shown). Finally, stimulated A-hTg was strongly and significantly correlated to both stimulated hTg at 8-12 months after radioiodine treatment and stimulated hTg at 36 months after radioiodine treatment (*P* < 0.001, *r* = 0.877, and *P* < 0.001, *r* = 0.853, respectively).

### 3.4. A-hTG and Combination with BRAF^V600E^ Mutation with Respect to ATA Classification

**Figure 2:**
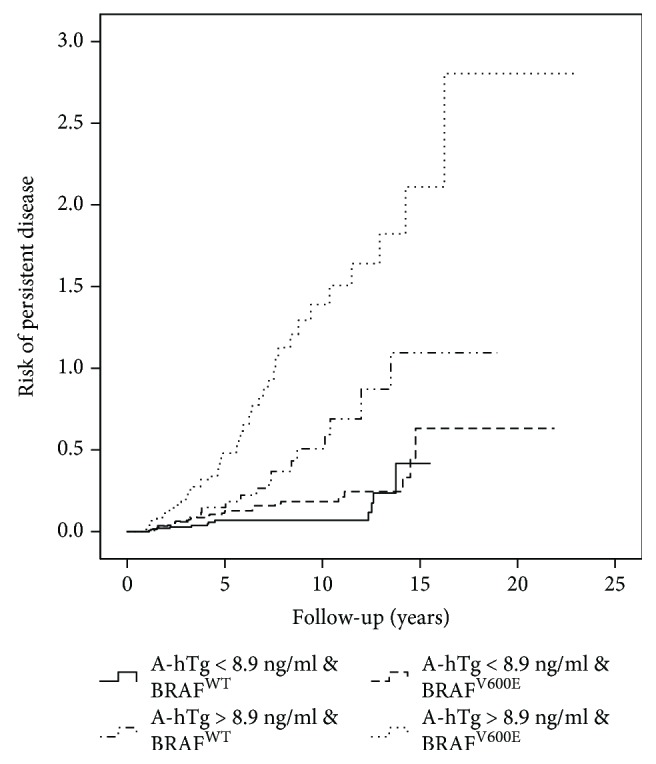
A-hTg and *BRAF* mutation predict persistent disease in all PTC patients.

By using the chi-square test, we found that the combination of stimulated A-hTg with *BRAF* status was significantly associated with PD in all PTC patients (*P* = <0.001) (Figure 2).

**Figure 3:**
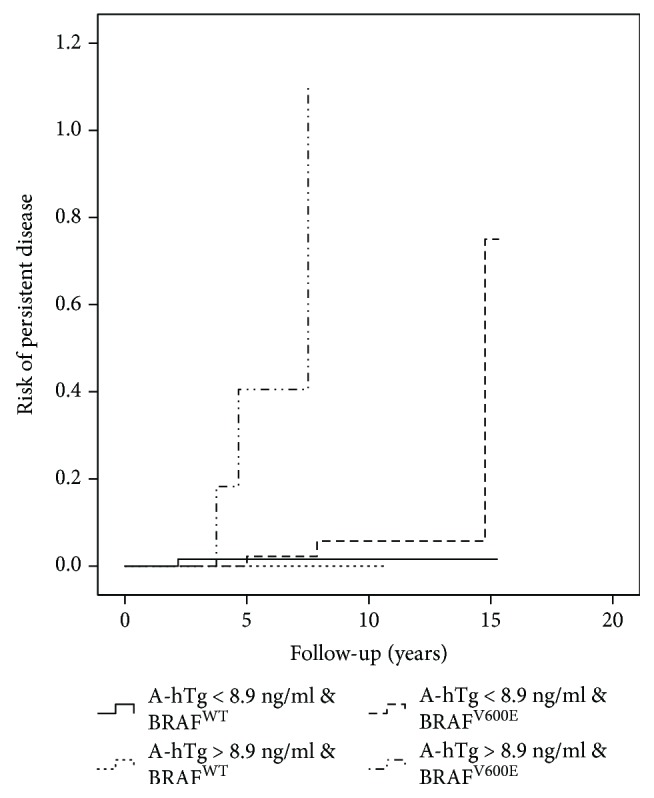
A-hTg and *BRAF* mutation predict persistent disease in the low-risk ATA classification.

**Figure 4:**
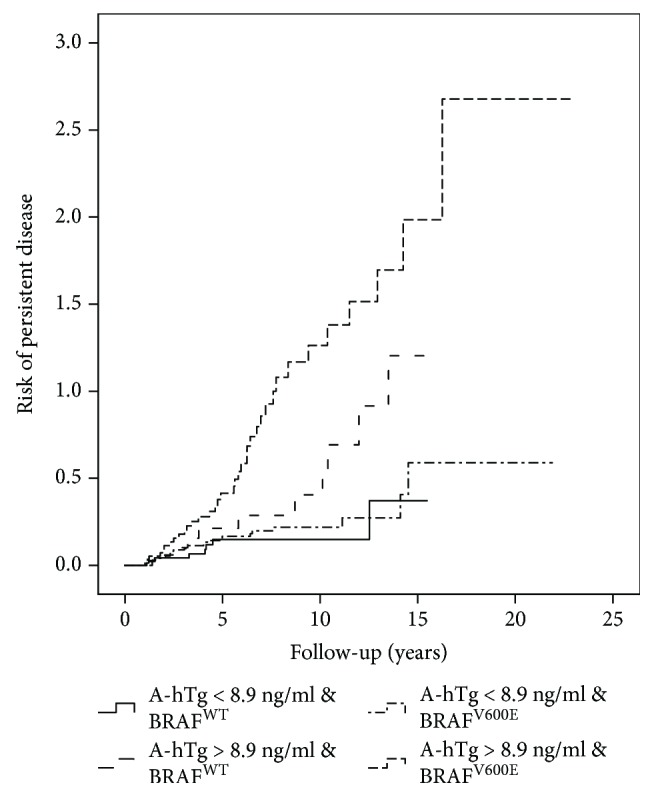
A-hTg and *BRAF* mutation predict persistent disease in the intermediate-risk ATA classification.

**Figure 5:**
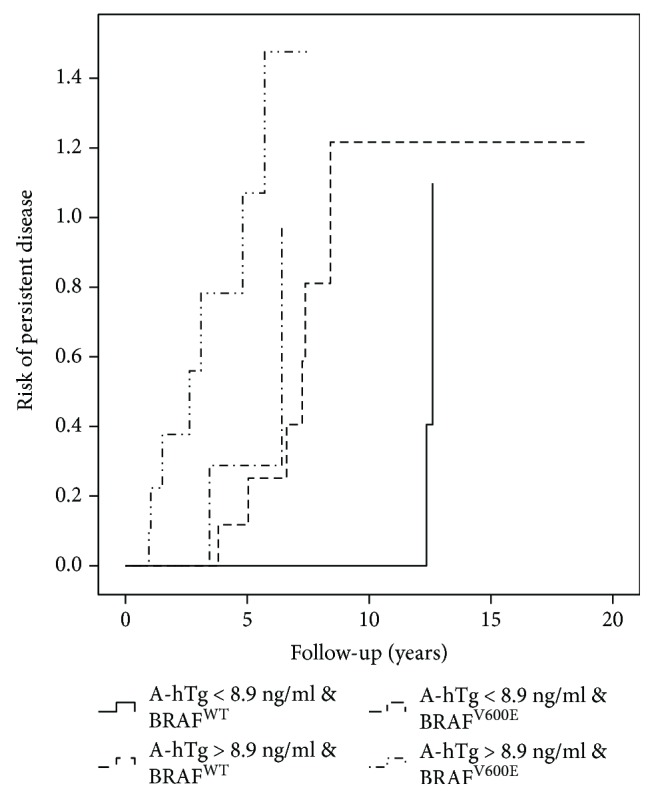
A-hTg and BRAF mutation predict persistent disease in the high-risk ATA classification.

As shown in Table 3 and in Figures 3–5, the combination of A-hTg and the *BRAF* status in the different ATA class risks might improve the risk stratification of PD.

## 4. Discussion

This is the first study that combines the BRAF^V600E^ mutation with stimulated A-hTg according to the ATA class risk. Our data on the BRAF^V600E^ mutation rate reflect those described in the literature [6]. In particular, we found that BRAF^V600E^ is an independent factor involved with the persistence of disease [6–11] and is associated with the tall cell variant, extrathyroidal spread, lymph node involvement, ATA intermediate-high-risk categories, and advanced stage at presentation. In effect, in the recent ATA Guidelines, *BRAF* mutation was included as a risk factor for structural disease recurrence [15]. Before the new ATA Guidelines, Prescott et al. have shown that *BRAF* status adds incremental value to ATA class risk [14]. Also, we confirmed that stimulated A-hTg was an important factor associated with persistent disease.

In our series, the stimulated Htg ablation cut-off level was calculated at 8.90 ng/ml. This value, similar to Melo et al.'s study (7.2 ng/ml) [17], represents a good marker of persistent disease, especially in terms of specificity, and is an unfavourable prognostic factor worse than the uptake at posttherapeutic WBS. In effect, *BRAF* mutation induces a reduction in I131 uptake in thyroid tumours, through several genetic and epigenetic mechanisms [22, 23]. In addition, it promotes NIS-silencing by histone deacetylation at critical regulatory regions of NIS promoters [24], thus reducing I131 uptake. Therefore, it is possible to identify a subset of thyroid cancer with poor uptake from the first radioiodine treatment by combining high A-hTg levels with absent or reduced uptake at WBS postradioiodine treatment. We did not find any significant difference regarding the performance of AUC cut-off of stimulated A-hTg ablation prepared by THW or Thyrogen in comparison with the work of Pitoia et al. [19] and Ciappuccini et al. [18]. In addition, the modality of preparation for radioiodine treatment (THW vs. Thyrogen) was not significant in the Cox regression analysis with respect to persistent disease. However, in our cohort, stimulated A-hTg strongly correlated with stimulated hTg at 8-12 months after radioiodine treatment anticipating the dynamic risk stratification.

Therefore, the combination of these two parameters (*BRAF* mutation and stimulated A-hTg) allows us to better discriminate patients at greater risk, depending on the ATA class risk. In particular, in the low-risk classification, only the combination of higher stimulated A-hTg and *BRAF* mutation was associated with persistent disease. In the intermediate-risk classification, higher A-hTg with or without *BRAF* mutation was associated with persistent disease; finally, in the high-risk classification, *BRAF* mutation regardless of stimulated A-hTg was associated with persistent disease.

The present study confirms the appropriateness of using the *BRAF* mutation and stimulated A-hTg to redefine the risk of persistence disease, according to the ATA risk class. It is obvious that dynamic risk stratification remains the best method to determinate the real risk of persistent disease in patients with DTC [25–27].

## Figures and Tables

**Table 1:** Clinical and pathological differences between the BRAF^WT^ and BRAF^V600E^ groups.

	BRAF^WT^ (*n* = 263, 42.4%)	BRAF^V600E^ (*n* = 357, 57.6%)	*P* value
Age (years)	51.5 ± 15.8	50.0 ± 15.4	0.261^A^
Age > 45 years	167 (63.5%)	213 (59.7%)	0.359^B^
Gender (F/M)	187/56	257/100	0.857^B^
Incidentally	97 (36.9%)	59 (16.5%)	<0.001^B^
Hashimoto's thyroiditis	112 (42.9%)	140 (39.7%)	0.455^B^
Tumour size (mean ± SD, cm)	1.74 ± 2.00	1.34 ± 0.99	0.003^A^
Tumour > 1 cm	144 (45.4%)	173 (56.4%)	0.143^B^
Multifocality	107 (40.7%)	197 (55.2%)	<0.001^B^
Histology			
Classic	85 (25.2%)	252 (74.8%)	<0.001^C^
FVPTC	110 (69.2%)	49 (30.8%)	
Tall cell	5 (14.7%)	29 (85.3%)	
T (T3-T4)	78/270 (28.8%)	192/270 (71.2%)	<0.001^D^
N1 (a+b)	56/181 (30.9%)	125/181 (69%)	<0.001^D^
Distant metastases	15 (5.7%)	7 (2%)	0.015^B^
R1-2	63/194 (32.4%)	131/194 (67.5%)	<0.001^D^
Extrathyroidal extension	78 (28.9%)	192 (71.1%)	<0.001^B^
Vascular invasion	49/104 (47.1%)	55/104 (52.9%)	0.328^B^
Stage (III-IV)	48/170 (28.2%)	122/170 (71.7%)	0.039^D^
Number of patients who underwent I131 treatment	219 (83.3%)	336 (94.4%)	<0.001^B^
mCi ablation			
30 mCi	34 (55.7%)	27 (44.3%)	0.001^D^
50 mCi	41 (47.1%)	46 (57.9%)	
100 mCi	144 (35.4%)	263 (64.6%)	
WBS postdose (metastatic uptake)	20 (9.3%)	19 (5.7%)	0.126^B^
A − hTg > 8.9 ng/ml	58 (42.3%)	79 (57.7%)	0.421^B^
ATA risk			
Low	123 (58.3%)	88 (41.7%)	<0.001^D^
Intermediate	123 (32.5%)	255 (67.5%)	
High	17 (54.8%)	14 (45.2%)	
Years follow-up	6.20 ± 4.13	6.07 ± 4.23	0.629
Recurrence	4 (1.6%)	16 (4.6%)	0.040^B^
Positive DRS	41 (15.6%)	87 (24.4%)	0.009^B^
Persistent disease	33 (12.5%)	82 (23%)	0.001^B^

^A^*T*-test; ^B^Fisher's exact test; ^C^chi-square test; ^D^linear-by-linear association test.

**Table 2:** Cox regression analysis (multivariate) for PD in all PTC patients.

PD at ablation time	Hazard ratios	CI 95%	*P* value
A-hTg > 8.9 ng/ml	2.762	1.824-4.181	<0.001
BRAF^V600E^	1.887	1.232-2.891	0.004
R	1.747	1.190-2.566	0.004
T	1.407	1.153-1.718	0.001
N	1.311	1.129-1.522	<0.001

PD: persistent disease.

**Table 3:** A-hTg values and BRAF status combination in the prediction of PD in all PTC according to ATA class risk.

ATA risk	A-hTg and *BRAF* status	Free disease	PD	*P* value	HR (CI 95%)
Low risk	A-hTG < 8.9 ng/ml & BRAF^WT^	79	1	N.S.	
	A-hTg > 8.9 ng/ml & BRAF^WT^	12	0	N.S.	
	A-hTg < 8.9 ng/ml & BRAF^V600E^	61	3	N.S.	
	A-hTg > 8.9 ng/ml & BRAF^V600E^	5	3	0.001	60.2 (5.28-687)

Intermediate risk	A-hTG < 8.9 ng/ml & BRAF^WT^	70	8	N.S.	
	A-hTg > 8.9 ng/ml & BRAF^WT^	25	12	0.029	2.71 (1.106-6.670)
	A-hTg < 8.9 ng/ml & BRAF^V600E^	158	28	N.S.	
	A-hTg > 8.9 ng/ml & BRAF^V600E^	25	36	<0.001	5.001 (2.318-10.790)

High risk	A-hTG < 8.9 ng/ml & BRAF^WT^	3	3	N.S.	
	A-hTg > 8.9 ng/ml & BRAF^WT^	3	6	N.S.	
	A-hTg < 8.9 ng/ml & BRAF^V600E^	1	3	0.042	5.963 (1.069-33.255)
	A-hTg > 8.9 ng/ml & BRAF^V600E^	2	8	0.002	11.564 (2.543-52.576)

N.S.: not statistically significant; PD: progressive disease; HR: hazard ratio.

## Data Availability

The data used to support the findings of this study are available from the corresponding author upon request.
